# microRNA-449a functions as a tumor-suppressor in gastric adenocarcinoma by targeting Bcl-2

**DOI:** 10.3892/ol.2013.1609

**Published:** 2013-10-09

**Authors:** BIN WEI, YING SONG, YONGHONG ZHANG, MINGJUN HU

**Affiliations:** 1 Department of Gastroenterology, Xi’an First Hospital, Xi’an, Shaanxi 710002, P.R. China; 2 Department of Gastroenterology, Central Hospital of Xi’an City, Xi’an, Shaanxi 710003, P.R. China

**Keywords:** miR-449a, Bcl-2, gastric adenocarcinoma, caspase 3, caspase 7, apoptosis

## Abstract

microRNAs (miRNAs or miRs) may function as oncogenes or tumor suppressors. The present study identified that miR-449a was downregulated in human gastric cancer. The overexpression of miR-449a inhibited gastric adenocarcinoma cell growth and promoted cell apoptosis in the MGC-803 and SGC-7901 gastric adenocarcinoma cell lines. Subsequently, Bcl-2 was identified as a potential miR-449a target by bioinformatics analysis. It was also shown that Bcl-2 was negatively regulated by miR-449a at the post-transcriptional level, via a specific target site within the 3′-untranslated region (3′UTR), by luciferase reporter assay. The expression of miR-449a induced cell apoptosis, as observed by TdT-mediated dUTP nick end labeling and caspase 3/7 assays, and was rescued by Bcl-2 expression. Therefore, these observations indicate that miR-449a acts as a tumor suppressor by targeting the Bcl-2 gene and that it promotes gastric adenocarcinoma cell apoptosis via Bcl-2. The findings of this study contribute to or current understanding of the functions of miR-449a in gastric adenocarcinoma.

## Introduction

Gastric cancer is the second most common and the leading cause of cancer mortality worldwide, with ~1,000,000 new cases per year (1). It is estimated that 50% of the cases occur in Asia, mainly in China (2). Previous studies have revealed several genes related to human gastric cancer (3,4), but successful therapeutic targets are limited. Thus, the molecular mechanisms of gastric cancer are of great importance and remain to be elucidated. Adenocarcinoma is the most common form of gastric cancer, and elucidating the mechanisms of its development will provide us with more knowledge to identify novel and promising agents for the cure and treatment of gastric cancer.

Recently, the classical oncogenes and tumor suppressors have been expanded to include a new family of RNAs known as microRNAs (miRNAs), as miRNAs have emerged as significant protein regulators via the direct interaction with complementary sites in the 3′-untranslated region (3′UTR) (5). miRNAs are able to regulate a broad spectrum of physiological and developmental processes, as well as cancer initiation and progression. Previous studies have identified cancer-specific miRNAs in numerous types of cancer, including cervical cancer (6), lung cancer (7), melanoma (8), colorectal cancer (9), breast cancer (10) and gastric cancer. The overexpressed or underexpressed miRNAs in cancers may function as oncogenes or tumor suppressors, respectively. It has been demonstrated that miRNA genes are frequently located in cancer-associated genomic regions or fragile sites (11). Furthermore, miRNA expression profiles provide a better classification of function for poorly-differentiated cancers compared with mRNA profiling assays (12). These facts indicate that miRNAs play a critical role in cancer.

Apoptosis, which is a form of programmed cell death, maintains the balance between mitosis and cell death in multicellular organisms (13). Once the regulation of apoptosis interferes with this balance of factors, the disruption may lead to cancer. Over the past decades, numerous oncogenes and tumor suppressor genes, including Bcl-2 and p53, have been identified that regulate apoptosis (14). Recently, miRNAs have been found to regulate cell apoptosis via these tumor suppressors or oncogenes (15,16). These studies have broadened our understanding of apoptotic signaling pathways and their dysregulation in cancer progression.

In the present study, we aim to explore the role of miR-449a in the adenocarcinoma cell lines, MGC803 and SGC-7901.

## Materials and methods

### Human cancer tissue samples

Fresh frozen human gastric adenocarcinoma tissue samples and matched normal gastric tissue samples were obtained from the Tumor Bank Facility of the First People’s Hospital of Xi’an (Xi’an, Shaanxi, China). The tumor types were confirmed by pathological analysis. All human materials were used in accordance with the policies of the Institutional Review Board. The study was approved by the ethics committee of Xi’an First Hospital (Xi’an, China). Written informed consent was obtained from the patients.

### Vector construction and luciferase reporter assay

In order to construct the Bcl-2 3′UTR plasmid, a wild-type 3′-UTR fragment of human Bcl-2 mRNA containing the putative miR-449a binding sequence was amplified by PCR and cloned downstream of the firefly luciferase gene in the pMIR-REPORT vector (Ambion, Life Technologies, Carlsbad, CA, USA) between the *Hind*III and *Spe*I sites. This produced the pMIR-Bcl-2-3′UTR luciferase vector (Bcl-2 3′UTR) using the following primer: Bcl-2 3′UTR sense, 5′-CGGACTAGTCTA TACATCCACAGGGTT-3′ and antisense, 5′-CCCAAGCTT TCTTTAGCCACTTCAGTT-3′. For the mutant reporter vector, seed sequences of miR-449a-binding sites in the Bcl-2 3′UTR fragment were mutated using the QuikChange Mutagenesis kit (Stratagene, La Jolla, CA, USA). The mutated Bcl-2 3′-UTR fragment was cloned into the pMIR-REPORT vector to develop the pMIR-Bcl-2-3′UTR-mut vector (Bcl-2 3′UTR-mut). For the luciferase assay in the MGC-803 cells, the cells were cotransfected in 48-well plates with Bcl-2 3′UTR or Bcl-2 3′UTR-mut, miR-449a or ASO-449a using Lipofectamine 2000 reagent. Luciferase activity was measured 24 h later by using a dual luciferase reporter assay (Promega, Madison, WI, USA) on a Fluorescence Spectrophotometer F4500 (Hitachi, Tokyo, Japan). The results were expressed as the relative luciferase activity (firefly luciferase/Renilla luciferase). All experiments were repeated three times in triplicate.

### Cell culture and transfection

Human gastric adenocarcinoma cell lines, MGC-803 and SGC-7901, were obtained from Shanghai Institute of Biochemistry and Cell Biology (Shanghai, China). The cells were maintained in RPMI-1640 (Gibco, Carlsbad, CA, USA) supplemented with 10% FBS and 1% penicillin/streptomycin, cultured at 37ºC in a humidified chamber supplemented with 5% CO_2_. Briefly, the cells were trypsinized, counted and seeded in plates the day prior to transfection to ensure a suitable cell confluence on the day of transfection. ASOs (Ambion, Life Technologies) were used at a final concentration of 200 nM and plasmids at 5 ng/μl, each in antibiotic-free Opti-MEM medium (Invitrogen). The transfection efficiency was monitored by Cy5-oligonucleotides.

### RNA preparation and quantitative (q)PCR

RNA extraction of the cells or tissue samples was performed with the mirVana miRNA Isolation kit (Ambion) according to the manufacturer’s instructions. Large RNA (>200 nt) and small RNA (<200 nt) were separated and purified by this procedure. The integrity of the large RNA was confirmed on 1% denatured agarose gel electrophoresis. For the detection of protein-coding genes, 5 μg large RNA extracted from the cells or tissue samples was reverse transcribed to cDNA primed by oligo(dT) using M-MLV reverse transcriptase (Promega). The cDNA was used for the amplification of the Bcl-2 genes. The PCR primers were as follows: Bcl-2 sense, 5′-CCGTTGGCCCCCGTTGCTTT-3′ and antisense, 5′-CTGGCGGAGGGTCAGGTGGA-3′ (17); β-actin sense, 5′-CGTGACATTAAGGAGAAGCTG-3′ and antisense, 5′-CTAGAAGCATTTGCGGTGGAC-3′. qPCR analysis was performed in triplicate with the SYBR Premix Ex Taq™ kit (Takara, Shiga, Japan), according to the manufacturer’s instructions, by initial denaturation at 94ºC for 4 min, followed by 40 cycles of amplification, using 94ºC for 60 sec, 58ºC for 60 sec and 72ºC for 60 sec for data collection.

### CCK-8 assay

The cells were plated in 96-well plates in 100 μl cell culture medium and incubated at 37ºC for 24 h. The cells were then transfected with miR-449a, ASO-449a or control oligonucleotides. Following incubation for 72 h, the cells were incubated with 10 μl CCK-8 (at a final concentration of 0.5 mg/ml) at 37ºC for 3 h. Subsequent to shaking for 20 min, the optical density was determined at 450 nm.

### Colony formation assay

Following transfection with miR-449a, ASO-449a or control oligonucleotides, the cells were trypsinized, counted and seeded for colony formation assay in 12-well plates. During colony growth, the culture medium was replaced every 3 days. The colony was counted only if it contained >50 cells, and the number of colonies was counted the 7th day after seeding. Each treatment was performed in triplicate.

### Apoptosis assay

Cell apoptosis was detected using the *In Situ* Cell Death Detection kit and fluorescein (Roche Applied Science, Indianapolis, IN, USA), which is based on TdT-mediated dUTP nick end labeling (TUNEL) technology. DAPI staining was used to determine the number of nuclei and to assess the gross cellular morphology.

For detection of caspase 3 and 7 activity, the cells were cultured in 96-well plates and treated with miR-449a or ASO-449a, and the Caspase-Glo^®^3/7 Assay (Promega, Mannheim, Germany), which is based on the cleavage of the DEVD sequence of a luminogenic substrate by caspases 3 and 7 resulting in a luminescent signal, was performed according to the manufacturer’s instructions (18).

### Western blot analysis

The cells were transfected with miR-449a, ASO-449a or control oligonucleotides and then were lysed with RIPA lysis buffer 72 h later and the proteins harvested. Following SDS polyacrylamide gel electrophoresis, the separated proteins were transferred onto a nitrocellulose membrane. The primary rabbit monoclonal antibodies to Bcl-2 (Santa Cruz Biotechnology, Santa Cruz, CA, USA) and GAPDH (SaierBio, Tianjin, China) were incubated with the blot overnight at 4ºC. GAPDH was used as an endogenous normalizer. Anti-rabbit IgG horseradish peroxidase-conjugated goat secondary antibody (SaierBio) was used and protein expression was assessed by enhanced chemiluminescence and exposure to chemiluminescent film. LabWorks™ Image Acquisition and Analysis Software (UVP) was used to quantify band intensities. All antibodies were purchased from Abcam (Cambridge, UK).

### Statistical analysis

The statistical analysis utilized a two-tailed Student’s t-test. Experimental results are expressed as the mean values ± SE. P<0.05 was considered to indicate a statistically significant difference.

## Results

### miR-449a is downregulated in human gastric adenocarcinoma tissues

**Figure 1:**
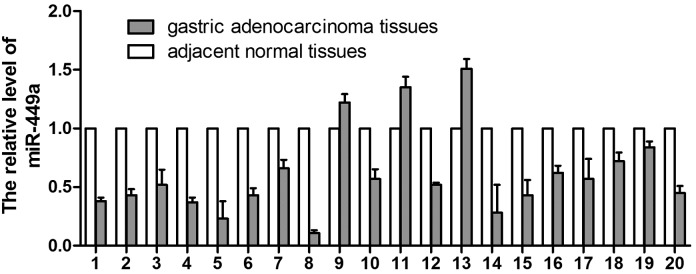
Identification of the differential expression of miR-449a in gastric adenocarcinoma tissues. The expression level of miR-449a in 20 pairs of gastric adenocarcinoma tissues and matched adjacent normal tissues were detected by quantitative (q)PCR. β-actin was used as endogenous normalizer. miR, microRNA.

In order to investigate the role of miR-449a in gastric adenocarcinoma, the expression levels of miR-449a were first measured in 20 pairs of human gastric adenocarcinoma tissues and adjacent normal tissues using qPCR. The results showed that the miR-449a expression levels were generally lower in the gastric adenocarcinoma tissues than in the matched normal gastric tissues, with the exception of three paired samples (Fig. 1).

### miR-449a inhibits gastric adenocarcinoma cell growth in vitro

**Figure 2:**
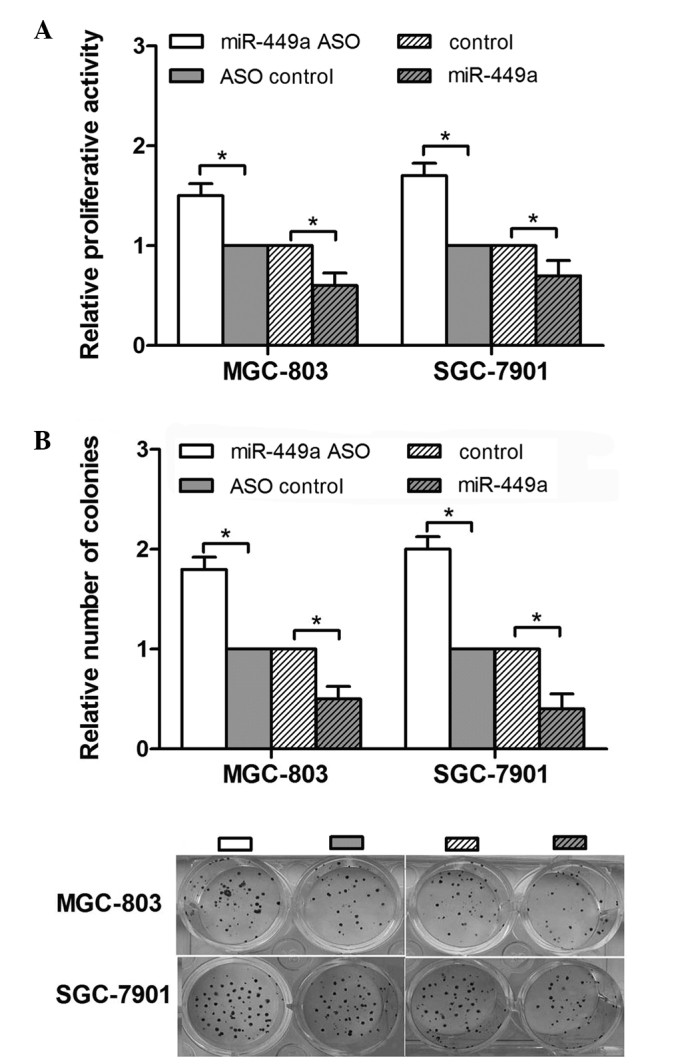
Alteration of miR-449a levels affects MGC-803 and SGC-7901 cell growth. (A) The cells were transfected with the miR-449a vector or miR-449a ASO. The CCK-8 assay was used to determine the relative cellular proliferation at 72 h. The cell absorption was measured at 450 nm. (B) Cell growth was also measured with the colony formation assay. The data represent the mean ± SD of three different experiments. ^*^P<0.05. miR, microRNA; ASO, antisense oligonucleotide.

To test miR-449a functions on gastric adenocarcinoma cell lines, the effects of altered miR-449a expression on MGC-803 and SGC-7901 cells were examined. Previous studies have confirmed that sequence-specific ASOs are able to inhibit miRNA activation (19), therefore, miR-449a ASO (ASO-449a), which was the exact antisense copy of the mature miR-449a sequence, was synthesized to inhibit the miR-449a function. MGC-803 cells were transfected with ASO-449a or control oligonucleotides. At 72 h post-transfection, the effect of miR-449a blocking on cell proliferation was evaluated by CCK-8 assay. In the MGC-803 cells, ASO-449a showed a significant antiproliferative effect compared with the control group (Fig. 2A), while overexpression of miR-449a inhibited cell proliferation in the gastric adenocarcinoma cells. The results in SGC-7901 cells were also confirmed. To further test the antiproliferative effect of miR-449a, a colony formation assay was performed. As shown in Fig. 2B, the colony number of the MGC-803 and SGC-7901 cells transfected with miR-449a ASO was significantly higher than those transfected with control oligonucleotides, while the cells transfected with miR-449a decreased significantly. These results indicated that miR-449a may be a tumor suppressor in gastric adenocarcinoma cells.

### Bcl-2 gene is negatively regulated by miR-449a by targeting putative binding sites in the 3′-UTR

Numerous putative miR-449a targets are predicted by various computer-aided algorithms known as PicTar, TargetScan and miR-Base Targets. Of the predicted target genes, the human proto-oncogene Bcl-2, whose mRNA 3′UTR contained a putative binding site of miR-9, was identified.

**Figure 3:**
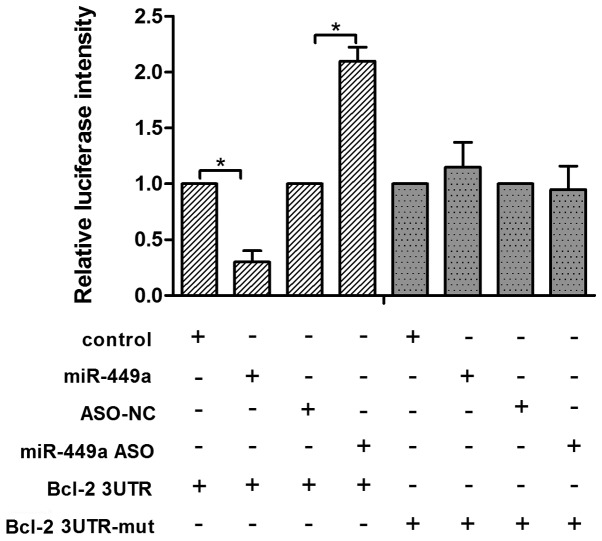
miR-449a directly targeting Bcl-2. The effect of miR-449a or miR-449a ASO on the luciferase activity of Bcl-2 3′UTR and Bcl-2 3′UTR-mut in which the putative miR-449a binding site was mutated is presented. miR, microRNA; ASO, antisense oligonucleotide.

To confirm that miR-449a binds to this region and causes translational repression, the luciferase reporter pMIR-Bcl-2-3′UTR was constructed. The MGC-803 cells were transfected with the reporter vector and miR-449a or ASO-449a. As shown in Fig. 3, the relative luciferase intensity in the Bcl-2 3′UTR + miR-449a group was significantly lower than that in the Bcl-2 3′UTR + pMIR-REPORT group, while Bcl-2 3′UTR + ASO-449a had a relative higher luciferase activity compared with the Bcl-2 3′UTR + ASO control group. Similarly, another luciferase reporter vector was constructed containing the mutational Bcl-2 3′UTR, and this showed that neither miR-449a nor ASO-449a was able to affect the luciferase intensity in this 3′UTR mutant vector. The results indicated that miR-449a is able to bind to the 3′UTR of Bcl-2 mRNA and repress gene expression. These data confirm the prediction that Bcl-2 is a direct target for miR-449a.

### miR-449a downregulates Bcl-2 mRNA and protein expression in gastric adenocarcinoma cells

**Figure 4:**
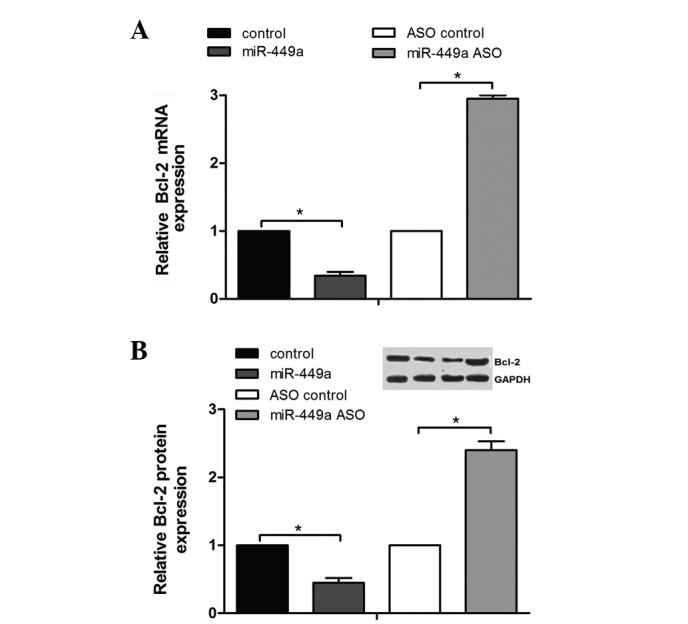
miR-449a inhibits Bcl-2 expression. (A) Quantitative (q)PCR shows that miR-449a is able to repress Bcl-2 mRNA expression. (B) Western blot analysis shows the suppression of the Bcl-2 protein by miR-449a, and the increase in the Bcl-2 protein level by miR-449a ASO. miR, microRNA; ASO, antisense oligonucleotide.

miRNAs suppress the expression of target genes through translational repression or degradation of a target transcript. Taking into account the above results, the present study assessed whether miR-449a had a functional role in the downregulation of endogenous Bcl-2 expression. The MGC803 cells were transfected with miR-449a to enhance its function, and the expression of Bcl-2 mRNA was measured by qPCR. As a result, when miR-449a was overexpressed, Bcl-2 mRNA was subsequently decreased by 0.6-fold compared with the control group, while the Bcl-2 cells transfected with ASO-449a showed a 2.8-fold increase (Fig. 4A), indicating that miR-449a regulates endogenous Bcl-2 mRNA levels via a mechanism of mRNA degradation. To further confirm the results obtained from qPCR, the expression level of the Bcl-2 protein was also analyzed. As shown in Fig. 4B, compared with control group, the Bcl-2 protein was downregulated in the cells transfected with miR-449a, and upregulated in the ASO-449a transfected cells, with an average 0.55-fold decrease and 2.6-fold increase separately, indicating that miR-449a is capable of inhibiting Bcl-2 expression through directly targeting Bcl-2 3′UTR, when taking into account the results in the previous section.

### miR-449a-repressed MGC-803 cell function is mediated by Bcl-2

**Figure 5:**
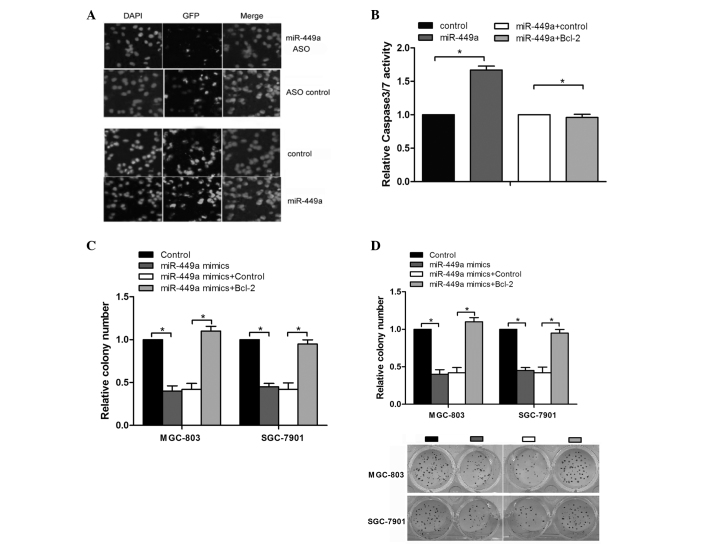
Effect of miR-449a on MGC-803 cell function is mediated by Bcl-2. (A) Overexpression of miR-449a induced TUNEL-positive cells and miR-449a ASO decreased TUNEL-positive cells. Green fluorescent protein (GFP) staining was used to determine apoptotic cells. DAPI staining indicated the number of nuclei to assess the gross cellular morphology (magnification, ×40). (B) Overexpression of miR-449a stimulated caspase-3/7 activity in MGC-803 cells and when co-expressed with Bcl-2, the increased caspase 3/7 activity was blocked. The results shown are representative of three independent experiments. (C and D) Cells were co-transfected with miR-449a mimics and Bcl-2 or control, then cell growth was analyzed with (C) MTT and (D) colony formation assays. miR, microRNA; TUNEL, TdT-mediated dUTP nick end labeling; ASO, antisense oligonucleotide.

A previous study reported that abnormal Bcl-2 expression is an important factor in the biological behavior of gastric carcinoma and that it is capable of regulating apoptosis (20). In order for miR-449a to downregulate Bcl-2 expression, we hypothesize that miR-449a may induce gastric adenocarcinoma cell apoptosis. Hence, a TUNEL assay was performed in the present study; a representative experiment is shown in Fig. 5A, which indicated that more TUNEL-positive cells were present in the cells transfected with miR-449a, while cells transfected with ASO-449a were attenuated for apoptosis compared to the control group.

Since apoptosis is ultimately mediated by caspase 3 and caspase 7, a Caspase-Glo 3/7 Assay was conducted. As shown in Fig. 5B, in the MGC-803 cells, the overexpression of miR-449a induced the luminescent signals of caspase 3 and 7, while the signal induced could be rescued in the cells by Bcl-2 expression. miR-449a also induced MGC-803 and SGC-7901 cell inhibition, which could be aborted by the overexpression of Bcl-2 (Fig. 5C and D). These results showed that miR-449a is able to repress cell proliferation and induce cell apoptosis, and that this effect may be rescued by Bcl-2 overexpression.

## Discussion

Gastric adenocarcinoma is one of the most highly lethal malignancies in the world (21). The disease is often detected at a late stage and the five-year survival rate is consequently at a low level, ranging between 5 and 15% (22). The incidence rate of gastric cancer is comparatively high in Eastern Asia. In 2002, the incidence rate of gastric cancer (0.74 million mortalities and 0.98 million new cases) ranked it as the second leading cause of cancer worldwide (23). The majority of gastric cancer cases occur in developing countries and there is a wide variation of gastric cancer incidence in different regions (24). To a certain extent, the low survival rates are due to the poor understanding of the mechanism of the cancer. It is therefore important to identify the mechanism that participates in the tumorigenesis of gastric adenocarcinoma.

It is assumed that underexpressed miRNAs in cancers may function as anti-oncogenes and vice versa (25). Given that miR-449a has a lower expression level in cancer cells, we conjectured that miR-449a may be a growth inhibitor in gastric adenocarcinoma. We predicted that the overexpression of miR-449a function may result in the arrest of growth. ASOs for miR-449a were used to enhance miR-449a function. In the CCK-8 and colony formation assays, it was shown that miR-449a inhibited MGC803 and SGC-7901 cell proliferation. To unveil the molecular mechanism of miR-449a in the regulation of cancer progression, Bcl-2 was experimentally identified as the direct target of miR-449a. Luciferase reporter assay-validated miR-449a targets the 3′UTR of Bcl-2, subsequently downregulating the endogenous level of Bcl-2, as observed using western blotting and qPCR analysis.

Bcl-2, an significant anti-apoptosis regulator located at chromosome 18q21 and encoding a 26-kDa protein localized mainly in the mitochondrial membrane (26), is overexpressed in gastric adenocarcinoma (27). This protein suppresses apoptosis through the mitochondrial pathway and subsequently enhances cell survival. The mitochondrial pathway includes the activation of pro-apoptotic factors, such as Bax, forming heterodimers and antagonizing the antiapoptotic effect of Bcl-2 (28), subsequently resulting in the activation of caspase 3 and 7. Therefore, the protein level of caspase 3 and 7 were detected in the present study and it was found that they were upregulated when cells were transfected with miR-449a. TUNEL assay confirmed that miR-449a is capable of promoting cell apoptosis.

p53, a significant apoptosis regulator, interacts with Bcl-2 family members at the mitochondria, involving the transactivation of Bax and the transcriptional repression of Bcl-2. p53 is able to directly activate the pore-forming functions of Bax (29). Recently a study identified that p53 is able to transcriptionally regulate Dicer, which is the miRNA processing complex, subsequently affecting the miR-449a expression level (30). Therefore, we speculate that another pathway may exist by which p53 regulates miR-449a, which then targets Bcl-2 and downregulates its expression. Further investigation into the circumstances under which p53 regulates Bcl-2 directly or through the miR-449a-targeting pathway may also be conducted. The clarification of this should allow us to further understand the role of miR-449a in gastric adenocarcinoma.

In conclusion, the present results show that miR-449a, an important anti-oncogenic miRNA associated with apoptosis, is downregulated in gastric adenocarcinoma. The enforced expression of miR-449a suppresses gastric cancer cell proliferation and induces apoptosis through directly targeting Bcl-2. These findings indicate that the frequently downregulated miR-449a in gastric adenocarcinoma contributes to gastric cancer proliferation, and that miR-449a may have a therapeutic potential in the suppression of gastric adenocarcinoma, therefore providing us with an improved understanding of the molecular mechanism of gastric adenocarcinoma initiation and progression.
